# On the Modus Operandi of the Poison of Venomous Reptiles, &c.

**Published:** 1848-09

**Authors:** 


*On the Modus Operandi of the Poison of Venomous Reptiles, &c.* By ALEXANDER MEANS, A. M., Professor of Chemistry and Pharmacy in the Medical College of Georgia, and Professor of the Physical Sciences in Emory College, Oxford, Ga.

The following article came under notice while referring to the back numbers of our exchanges in order to discover what had been communicated with respect to the treatment of poisoning by the bite of a rattlesnake. This examination was suggested by the interesting article by our esteemed correspondent, Dr. Trowbridge, contained in the original department of this number of our Journal. In connection with that article, we have thought that the essay by Dr. Means would be acceptable to our readers. It was published in the *Southern Medical and Surgical Jonrnal* for January, 1846.—EDITOR BUFFALO MED. JOUR.

"The brief disquisition which follows, was called forth in reply to some interesting inquiries received by letter from an intelligent young friend ardently engaged in the study of Medicine, and who had for some time been prosecuting a series of experiments upon the virus of serpents.

The article, therefore, was not written for the Journal, but at the special request of some medical friends, it has been hesitatingly submitted for publication, with the humble hope that the views which it presents, may aid, at least some of the *younger* members of the profession, in forming correct opinions upon the mode of action and pathological effects of animal poisons—an important pre-requisite to the administration of judicious and appropriate remedies.

First, then: How do poisons act upon the human system, (especially the poison of serpents,)—is it through the medium of the *blood*, or from impressions made upon the sentient extremeties of the *nerves*, and communicated along their tissue to the several nervous centres? So late as twenty years ago, in contravention to the doctrines of "the humoral pathology," Professors in the *first* medical schools in the United States, taught that *morbific*, as well as *sanative* impressions were propagated *exclusively* by *sympathy*, from one point to another of the human system; nor would they even allow the necessary intervention of the nervous tissue, as the *Odos* along which these impressions were communicated. More sound physiological views now generally obtain, but there are yet many medical philosophers, who reject the doctrine of *venous absorption*, and regard all impressions as communicated by nervous impulse. The most modern of those who have presented a plausible defence of these views, accompanied by appropriate (?) experiments, are Addison and Morgan, in an "essay" published in London in 1829, "on the operation of poisonous agents." These gentlemen, however, only deny that absorption is *in any case necessary* to the operation of poison, and yet contend vehemently against the action of medicinal agents *at all* through the medium of the circulation. A distinguished Professor in the medical department of the Pennsylvania University, has long been an uncompromising unbeliever, and an exclusive solodist of the old school, and so far as is known to the writer, has never, to the present hour, publicly announced any change in his views. Dr. Charles Caldwell, of the West, has also, for more than twenty years, as well in the lecture room, as from the press, warmly and inflexibly vindicated similar opinions. Now, we hold the doctrine of Imbibition to be so well settled at the present day by the experiments of Magendie, Muller, Blake, Mitchell and others, as to challenge skepticsm itself. But to the question more directly. Do poisons act by this process? 1st. It is known that whatever promotes absorption, hastens the action of medicines. 2nd. Almost every medicinal and poisonous substance, whose sensible or chemical properties subject it to ready detection, has been discovered in the blood or some of the secretions formed from it—and this too after the ordinary modes of administration. But the rapidity with which some deleterious agents act, has given rise to the opinion that these morbific impressions could not have been made through the circulatory system.

Now from Mr. Blake's experiments, half a drachm of concentrated hydrocyanic acid (one of the most virulent and diffusible poisons in the world) when poured upon the tongue, produced no morbid symptom until after the lapse of *eleven* seconds—death ensuing in *thirty-three* seconds after its administration, and this too when the fatal result was no doubt hastened by the inhalation of the vapor and its diffusive contact with the mucus lining of the air cells, which, according to Leibig's recent experiments, constitutes the most speedy and effective mode of action for this poison. Whereas by the result of satisfactory experiments by the same gentleman, (Dr. B.) Prof. Herring, of Stutgard, &c., it is ascertained that a substance will pass from any part of the vascular system in *dogs*, back again to the same point, in from *twelve* to *twenty seconds*. Now poison, it is believed, does not act in less than "*nine seconds*" (and this limit we think too small) after its introduction into the capillaries or veins, and therefore there is sufficient time for the poisonous molecules to pass to the various organs and tissues by the blood. Again: the manifestation of poison from the bites of venomous reptiles, in all the cases which I have known, or of which I have read, do not occur in less time than this, and may therefore be supposed to act through the venous channels.

One of the worst cases which occurs to my recollection, and which originated from the bite of a viper, is that described by Dr. Braun, and reported by Dr. Christison in his work on poisons, where a professed snake-charmer attempted to show that he could thrust the head of the animal into his mouth with impunity,—he introduced it, but suddenly dashed it from him with violence, when it was ascertained "that he was bitten near the root of the tongue." Here, where the part was so *vascular*, so *moist*, and so liberally supplied with *nerves*, it was not until "a few minutes afterwards," says the Doctor, that "he became so faint that he could not stand, and died within fifty minutes." This case, therefore, allowed sufficient time for venous transmission.

Before we abandon our enquiries upon this point, it is proper to remark that the distinguished author from whose valuable and popular treatise the above case has been extracted, and who at one time was inclined to favor the doctrine of the sympathists in accounting for the diffusion of poisonous impressions, has evidently felt himself somewhat embarrased in the maintainance of those views, since the experiments of Mr. Blake upon hydrocyanic acid, and although, at the time in which his last edition was ready for the press (Nov. 1844) he was confessedly not convinced that this virulent poison always acts by absorption, yet, with the ingenuousness characteristic of a great mind, he remarks, "on a dispassionate view of the whole investigation, it must be granted to be doubtful, whether this argument" (i. e., in favor of a sympathetic action,) "can now be appealed to in its present shape with the confidence which is desirable. And on the whole, the velocity of the circulation on the one hand, and the celerity of the action of certain poisons on the other, are both of them so very great, and the comparative observation of the time occupied by the two phenomena respectively, becomes in consequence, so difficult and precarious, that it seems unsafe to found upon such an inquiry, a confident deduction on either side of so important a physiological question, as the existence or the non-existence of an action of poisons by sympathy." The discovery of Dr. Herring of Stutgard, made a few years before, and reported by Dr. Christison himself, had shaken "the validity of many, though not all, of the facts which had been previously referred to the agency of nervous impression, on the ground of the celerity with which the effects of poisons are manifested." In this case it was shown that "the ferrocyanide of potassium, injected into the jugular vein of a horse, was discovered throughout the venous system at large in the short space of *twenty* or *thirty* seconds, and consequently must have passed in that period throughout the whole double circle of the pulmonary and systemic circulation." The vast improvements in chemical analysis within the last few years, (some of them, since the first publication of Dr. C's excellent work,) and employed by toxicologists in the detection of poisonous agents, circulating in the blood, have contributed much to throw light upon this hitherto obscure region of physiological research. These discoveries, together with very recent toxicological experiments, some of them even since the publication of the *last* edition of the treatise referred to, incline us but the more confidently to maintain the views above advanced, viz: that to venous transmission, and not to sympathetic action, we are to refer the morbid phenomena which result from the introduction of poisons into the human system. Among the most recent experiments furnished from any quarter are those of Dr. H. Meyer, upon the effects of prussic acid, and reported in the October number of the British and Foreign Medical Review. He confirms the correctness of Professor Emmert's experiments, to which we may hereafter refer, and states that "he found it to act *only* when received into the vascular system. On mechanically arresting the circulation, the poison did not act, although the integrity of the nervons system was preserved. On restoring the circulation, the operation of the poison was immediately observed." And again, as regards the lapse of time which intervenes between the introduction of the poison and its deleterious effects, he adds: "Hydrocyanic acid does not act so rapidly as it was formerly believed. Its operation was *never* instantaneous." The fallacy of the prevalent opinion that the deadly effects of this formidable volatile compound are experienced *immediately*, or at most within a *few seconds*, after its administration, so as to allow no time for acts of volition or locomotion, has been repeatedly proved.

A case, first reported in the Lancet of June 7th, of the present year, and again furnished in the last number of the Medical Review, is strikingly illustrative of the fact that there is time for freedom of thought and deliberate action, even after the largest draught of the poison, before its paralyzing effects are experienced. "A girl swallowed an *ounce* of prussic acid, recorked the phial, thrust the bottle to a full arm's length between the feather bed and the mattrass, got into bed, and then drew the clothes over her body." Again, this "protraction of symptoms" is still more satisfactorily evinced in a case reported by Mr. Godfrey, in the Provincial Medical and Surgical Journal, and copied into the Review: "A gentleman swallowed *half an ounce* of prussic acid, placed the bottle in the grate, walked to the top of a flight of stairs, (ten paces) descended the stairs, seventeen in number, and proceeded to a druggist's shop, (forty-five paces) making a total of fifty-five paces and seventeen stairs. He entered the shop in his usual manner, which was slow and easy, and said in his usual tone of voice, 'I want some more of that prussic acid'—his eyes then became fixed with a stare, he fell and died (probably) within ten or twelve minutes from the time of taking the poison." In fact, the violence of the action of this fatal drug has generally created such alarm and confusion in cases where it has been taken, that the celerity of its effects, although fearfully rapid in any event, has probably been exaggerated. From the very infrequency of its former administration, perhaps, these overwrought estimates of its speedy fatality required some time before they could be corrected. And nothing but careful observation, and a comparative familiarity with the alarming results of its action, could have enabled the medical philosopher deliberately to mark the progress of the pathological phenomena which it exhibits, and which are so readily traceable to its absorption and diffusion through the venous circulation. This being the most prompt and formidable article under the whole survey of toxicology, we have dwelt the longer upon its action, because if its rapidly occurring symptoms allow sufficient time for the vascular transmission of the absorbed poison, then we need entertain no scruples upon that head in regard to the *modus operandi* of the most intensely active virus found within the limits of the animal kingdom.

But *secondly:* If poisons act through the blood, how is the action propagated? I answer, it may be—1st, by maintaining its identity in the circulation, and thus being brought in contact with the structure of the organ or organs which it affects. Or, 2ndly, by acting chemically upon the constituents of the blood, and thereby so altering its vital condition as to unfit it for the purposes of life. To consider the *first* mode then. It is found by experiment that tartar-emetic, castor oil, strychnia, opium, and many other articles whose specific effects upon the stomach, alimentary canal, nerves, &c., when administered *per orem*, are well known, manifest precisely the same properties, and are directed in their action to the same organs or tissues, when *injected* into the *veins*,—proving, that their introduction into the circulation did not deprive them of their identity, and of their capability to excite their accustomed functional changes upon the various organs, most susceptible of their several specific impressions. Why, the medicinal molecules of tartar-emetic, when diffused throughout the mass of the circulation, should *prefer* to manifest their effects upon the *stomach*, rather than the lungs, the kidneys, or the salivary glands, is a question which has proved the sarcasm of a popular American writer,* and yet the astuteness of intellect which characterizes the Philadelphia Professor, would *surely* find no more difficulty in allowing such preference than in allowing the well established philosophical fact, that while *Light* is pouring upon the *ear*, the *palate*, and the *nostril*, with an intensity equal to that with which it falls upon the *eye*, yet the latter organ alone is sensitivé to tis action;—BECAUSE *neither the auditory, nor gustatory, but the visual nerve exclusively, was constituted to depend for the performance of its important function úpon the peculiar stimulus of that one agent, and the whole mechanical organism of the eye was designed for its admission and made subject to its laws.* In fact, every organ and every tissue has its own susceptibilities to impression, and the appropriate qualities must exist in the ágents employed to act upon it, before the characteristic developments of its nature can be expected.

By this sort of *physical election*, it would seem, the poisonous alkali, *conia*, acts upon the nervous system violently, by destroying its irritability and inducing paralysis. Probably the poison of some venomous serpents, sent rapidly by the torrent of the circulation to the *heart*, paralyzes that muscle, and occasions almost instant death, in accordance wïth the conclusions drawn by Dr. Meyer from his emperiments with hydrocyanic acid. Toxicological researches authorize the belief that poisons, by thus coming in contact with organic structures may derange functions or effect injurious and even fatal lesions. And if Magendie's observations are to be credited, even so bland, innocuous an agent as *atmospheric air*, when introduced into a vein, may, by its mechanical admixture with the blood-globules, so interrupt the pulmonary circulation as to produce asphyxia and death.

But to pass to the consideration of the *second* mode, i. e.—by *chemical combination.* And here let me premise what follows by remarking, in the language of Leibig,† that "no other part of the organism can be compared to the *blood*, in respect to the feeble resistance which it offers to exterior influences. The blood is not an organ which *is formed*, but an organ in

*Sec Chapman's Therapeutics, fourth edition, vol. 1, p. 73.

†Leibig's Chemistry in its application to Agriculture and Physiology.

the *act of formation;* indeed it is the *sum* of *all* the organs which are being formed. The chemical force and the vital principle hold each other in such *perfect equilibrium,* that every disturbance, however trifling, or from whatever cause it may proceed, effects a change in the blood." A great variety of substances are known by chemical action to cause signal changes in this fluid—as well acids and alkalis, as metallic salts and alcohol, &c. These substances perhaps mainly act upon the fibrin and albumen, and upon the constituents of the blood disks. *Hydrocyanic acid* unquestionably changes the *consistence* and *color* of the *blood,* and exhales its characteristic *peach-blossom odor* from almost all parts of the body, early *after* death, and even *before* through the *breath*—especially from the serous membranes, and has been detected by Meyer in the form of *cyanide* of *potassium,* not only in the blood, but also in the serous secretions and sundry soft solids. Oxalic acid, acetates, citrates and tartrates, are satisfactorily known to undergo decomposition in the course of the circulation. But I find myself involuntary overrunning my intended limits, and must withhold. From what has been said, however, we feel warranted in believing that some poisons act by forming compounds with the constituents of the blood which incapacitates it for vital purposes.

While, therefore, we could hardly feel justified in unequivocally denying the sympathetic action of *all* poisonous agents, through the nervous tissue alone, yet we must regard the burthen of facts and experiments as opposing the existence of such action, while their *venous distribution* has been established in many instances, *beyond question.* Emmert and Christison (and their experiments extended still farther by Blake) have *both* found that some of the most virulent poisons " have no effect whatever when the circulation of the part to which they are applied has been arrested." Again, they will not act upon a dissevered nerve, (though it may still retain its *irritability,*) when that nerve is isolated from the circulation. Nor will poison introduced into the largest blood-vessels, around which a ligature has been thrown in advance, act *at all,* (though the nerves of the part are all left *entire,*) until the ligature is removed—it then acts rapidly. Lastly, (upon this point,) Magendie, Brodie, Emmert and others, have tested the *quick, full,* and characteristic effects of poison by injection into the blood-vessels, when all the nerves supplying the part had been previously divided. Once more: If *nervous* influence propagated poisonous impressions, then surely as the *cutaneous* surface is covered by a perfect, unbroken net-work of nervous tissue, so continuous and extended that a pin's point cannot be laid down without striking a nerve, then a superficial snake-bite, where the fangs of the animal have but freely scratched the part and lacerated a great many nervous fibrillæ, ought to prove the most dangerous, as furnishing so many more points of radiation for the virus, whereas it is the fact that such bites are comparatively harmless, while those in which the fangs have penetrated deeply, as in the finger or foot, —where the part could be included within the jaws of the reptile, and the poison was injected in the neighborhood of deep-seated blood-vessels, have been uniformally most fatal.

And now, to your next enquiry:—Is Orfila right when he recommends *sleep?* If the views which I entertain of the pathology of such cases be correct, his counsel in this particular is certainly injudicious. My own conviction is that, like opium, conia, foxglove and tobacco, the virus of serpents deserves to be classed with the *cerebro-spinants* of Pereira, or those agents which excite the cerebral and true spinal system of nerves,—acting as powerful sedatives in reducing the force of the circulation, and either paralysing some portion of the nervous tissue by which the vital functions of the part are immediately suspended, (as, for example, the action of the *heart* or the *respiratory muscles*,) or substracting from its general healthy tone, and exciting irregular, spasmodic movements.

Mark the symptoms caused by the bite of the viper, (the only poisonous serpent known in Great Britain,) as detailed by Dr. Christison,* and compare them with some produced by the class of agents above referred to—viz: "lancinating pain, which begins in from three to forty minutes after the bite, and rapidly stretches up the limb—swelling, at first firm and pale—afterwards, red, livid and hard,—*tendency to fainting—bilious vomiting*—sometimes *convulsions*, (more rarely, jaundice,)—*quick, small, irregular pulse—difficult breathing—cold perspiration—dimness of vision* and *injury* of the *mental faculties*." Now the cardiaco-vascular sedatives, fox-glove and tobacco, "produce *nausea*,—sometimes *vomiting* and *purging—weakness* and *irregularity* of *pulse, syncope, impaired vision—giddiness* and *confusion of ideas:* while *paralysis, convulsions, delirium* and *stupor*, are occasional symptoms." The strong coincidence of symptoms will at once be recognized by comparing the words in italics. It is also a singular and interesting fact, in accordance with our view, that loss of intellect and sensation, *with* convulsions, generally result alike from the use of *hydrocyanic acid*, from *epilepsy*, and from *excessive hæmorrhage*, and that in *all these cases, ammonia* is a valuable remedy. Does not this argue a *oneness* of pathological condition to which the same remedial agent is applicable? Now that condition in *hæmorrhage* we know to depend upon an *atony* of the nervous system induced by the *sudden abstraction* of too great a quantity of *blood*—the natural stimulus of that tissue: and it may be legitimately inferred, therefore, in the other two cases. Why not also in cases of poisoning by serpents—where the *same restorative* (ammonia) is so successfully resorted to? The rationale of its operation we design to consider presently. But can *sleep* be advisable with these facts before us? We are again constrained to answer, No. Indeed, from our views of the character of the poison, and the superinduced condition of the system, we can but believe it would contribute to hasten a fatal termination. An atony of the nervous system has already been produced: it is *below par* in its functional capabilities, and, as the effects of *cold* and *opium* which overcome nervous sensibility, must be arrested by *rousing stimulants*, even amounting, in some cases, to the use of violent mechanical force; so, we apprehend, the influence of this class of sedative poisons, should be counteracted by *forced wakefulness* and *stimulants*. Every reader of history is familiar with the fact, that in crossing the frozen Alps, on their return from Egypt, the soldiers of Bonaparte were sinking by hundreds under the contra-stimulant effects of *cold*, and were only preserved by *flogging*, from falling into the *sleep* of *death*. How, then, may a combination of alcohol and ammonia (say in the form of brandy and hartshorn) operate favorably as an antidote to these poisons? I suppose that without resorting to a solution, dependent upon the chemical change in the substances, (which

* See Christison on Poison, vol. 2, p. 485.

we are satisfied, in some other instances *does* go on,) we may rationally account for their antidotal effects, by remembering that according to the best authorities in Pharmaco-dynamics, the specific region of action for *alcohol* is the *cerebrum* and *cerebellum*, while *ammonia* manifests its power mainly upon the *true spinal* or *excito-motory* system of nerves. Here, then, by the combination, we reach the nerves of the *encephalon* and *spinal marrow both.* May it not be, therefore, by this *extensive* rally upon the partially exhausted irritability of the nerves through the great thoroughfare of the sanguiferous channels, that these dreadful sedatives are counteracted. *En passant*, let me not forget to *urge*, in such cases, the propriety of inhaling as freely as possible the *vapor* from *diluted* aqua ammonia—(the concentrated water of the shops is too irritating and may produce troublesome or even dangerous inflammation of the mucous lining of the air passages.) The restorative salt, *carbonate* of *ammonia*, furnishes also a good form for inhalation. In both cases the minutely divided particles of æriform ammonia comes in extensive contact with the *whole mass* of blood circulating through the lungs, (the membranous coverings of the air-cells being readily permeable by gaseous bodies,) and consequently its characteristic effects are more promptly and decidedly manifested. But to conclude. The poisonous injections from the stings of insects, such as the wasp, the bee, the hornet, &c., may be regarded, we apprehend, as acting under some of the laws already considered.

Our opinions in regard to the pathology and *modus medendi* of such cases, may be briefly enunciated, however, as follows, viz:—The injected virus, by its powerful contra-stimulent effect, is supposed to produce a temporary spasm or paralysis of the capillary tubes, incapacitating them from conveying red globules, within the reach of its action, which is generally defined by a circumscribed, colorless areola, surrounding the point of puncture, but as the poisonous deposite is small—is introduced superficially, and is consequently limited to a small circle, the vitality and vigor of the neighboring circulation at length overcomes the morbid inaction, and an over-excitement takes place in the form of cellular inflammation, which, when uninterrupted, always ends in resolution. But, as in a case of superficial *pinch* or *bruise*, followed by whiteness of the part and injury of the subcutaneous vessels, it is known that immediate mechanical irritation, in the way of friction, will sustain and recruit the crippled and languid capillaries, from which the blood has been violently expelled, and enable them forthwith to transmit their usual contents,—thus at once restoring the circulation of the part and preventing *ecchymosis*:—so, by a process *not greatly* dissimilar, we suppose that the prompt and peculiar action of *ammonia* instantly overcomes the hyposthenic effect of the poison—restores tone to the *nervous* and *vascular* systems, and prevents subsequent inflammation. I have seen strong tincture of *camphor* almost instantly relieve the sting of a wasp; and although I have never tried it, see no good reason why sulphuric ether, liberally applied and *retained on the part*, might not prove a quick and valuable auxiliary to our list of antidotes.

